# Perspective: The Lung, Particles, Fibers, Nanomaterials, and Autoimmunity

**DOI:** 10.3389/fimmu.2020.587136

**Published:** 2020-12-18

**Authors:** K. Michael Pollard

**Affiliations:** Department of Molecular Medicine, The Scripps Research Institute, La Jolla, CA, United States

**Keywords:** lung, autoimmunity, particle, nanoparticle, fiber

## Abstract

Studies have shown that a wide range of factors including drugs, chemicals, microbes, and other environmental agents can induce pre-clinical autoimmunity. However, only a few have been confidently linked to autoimmune diseases. Among these are exposures to inhaled particulates that are known to be associated with autoimmune diseases such as lupus and rheumatoid arthritis. In this article, the potential of particle, fiber, and nanomaterial exposures to induce autoimmunity is discussed. It is hypothesized that inhalation of particulate material known to be associated with human autoimmune diseases, such as cigarette smoke and crystalline silica, results in a complex interplay of a number of pathological processes, including, toxicity, oxidative stress, cell and tissue damage, chronic inflammation, post-translational modification of self-antigens, and the formation of lymphoid follicles that provide a milieu for the accumulation of autoreactive B and T cells necessary for the development and persistence of autoimmune responses, leading to disease. Although experimental studies show nanomaterials are capable of inducing several of the above features, there is no evidence that this matures to autoimmune disease. The procession of events hypothesized here provides a foundation from which to pursue experimental studies to determine the potential of other environmental exposures to induce autoimmunity and autoimmune disease.

## Introduction

Numerous drugs, chemicals, microbes, and other environmental factors have been identified as possible causes of pre-clinical autoimmunity (1). However, very few have been confidently associated with human autoimmune diseases (2). Among these are respirable particulates such as cigarette smoke and silica dust (2) which have been linked to systemic lupus erythematosus (SLE), rheumatoid arthritis (RA), and systemic sclerosis (SSc) (2, 3). Asbestos, a fibrous silicate mineral, has also been associated with autoimmunity, although linkage to specific autoimmune diseases is less well established (2, 4). In contrast, a role for nanomaterial [materials with at least one dimension between 1 and 100 nanometers (5, 6)] exposure as a causative agent in human autoimmunity remains to be established.

In reviewing the etiopathogenesis of environment-induced autoimmunity, we (7) proposed that the toxic insult of an environmental exposure initiates a multi-step process characterized by tissue damage, and the release of nucleic acids and other damage associated molecular patterns (DAMPs) including self- and modified self-antigens, which induces an innate inflammatory response, leading to adaptive immunity involving the presentation of self- and modified self-antigens to non-tolerant lymphocytes. Included within this process is the engagement of Toll-like receptors (TLRs) and other innate sensors, the production of inflammatory mediators, the expansion of autoreactive B and T effector cell populations, and the production of autoantibodies. These events often occur at the site of exposure, where they can lead to the development of tertiary lymphoid structures (TLSs), and/or germinal centers in secondary lymphoid organs draining the site of exposure. In either case the expansion of autoreactive lymphocytes and their migration to target tissues, such as the kidney in SLE or the joints in RA, results in autoimmune disease (7).

To include an additional perspective to the above hypothesis, this article compares the current state of knowledge regarding the response of the immune system to selected particle, fiber, and nanomaterial exposures. Specific particulate and fibrous materials are discussed because of their known role in pre-clinical autoimmunity and autoimmune diseases (2). This is contrasted with the immunological responses to nanomaterials, particularly engineered nanoparticles (ENPs), where evidence for induction of autoimmunity is less convincing.

## Particulate Material

### Cigarette Smoke

Cigarette smoke is a complex mixture of particulate-phase and chemical compounds (8, 9), and is a significant risk factor for chronic obstructive pulmonary disease (COPD) (10). Smoking is also a significant risk factor for RA (11), and has been linked to other autoimmune diseases including SLE, multiple sclerosis, and Crohn’s disease (2). The linkage of smoking and RA is strongest for seropositive disease particularly patients with anti-citrullinated protein antibody (ACPA) (2) which is itself strongly linked to the HLA shared epitope (12). The association between smoking, RA, and the HLA-linked ACPA response identifies an important gene-environment interaction for autoimmune diseases (13).

The particulate material of cigarette smoke consists of partially combusted plant material between >0.25 to <1 um in size (8, 9, 14). The chronic inflammation resulting from cigarette smoke exposure is thought to be due to this particulate material and gas-phase chemicals but may also involve bacterial and fungal contaminants of tobacco (15). Cigarette smoke also contains reactive oxygen species (ROS), that may contribute to post-translational modification of proteins and other markers of oxidative stress (16). Tissue damage from inhaled smoke leads to activation of lung epithelial cells and alveolar macrophages (AM) *via* interaction between DAMPs and pattern recognition receptors (PRRs) such as TLRs (16). This initiates an innate immune response, characterized by inflammasome formation and the production of IL-1β, IL-18, and other proinflammatory mediators including IL-6 and TNF-α (16). The resulting infiltration of neutrophils and monocytes further exacerbates oxidative stress and inflammation. Additional processes, such as neutrophil death, mediated by NETosis, also leads to cell and tissue damage (17).

The innate inflammatory response progresses to adaptive immunity consisting of T helper cell responses, including Th1 and Th17 (18, 19), and B cells that can form lymphoid aggregates in the lung (10). These structures are similar to TLSs found in target organs of autoimmune diseases where they are argued to provide a microenvironment for the survival of autoreactive lymphocytes (20, 21). The presence of plasma cells in lung TLSs (21) suggests that the lungs are a source of autoantibodies, and this is supported by the presence of autoantibodies in bronchoalveolar lavage (BAL) fluid (21, 22). Moreover, autoantibodies to modified self-antigens have been described in COPD (21, 23, 24) including autoantibodies known to be important in seropositive RA [rheumatoid factor (RF) and ACPA] (11, 25). These events may occur years before clinical diagnosis of RA (26). It has been hypothesized that localized chronic inflammatory processes in the lungs following smoke exposure are important in the pre-clinical phase of not only diseases such as COPD but also autoimmune diseases such as RA (25, 27). Importantly, experimental studies have shown that TLSs and autoantibodies persist after ceasing smoking and are dependent upon IL-1 receptor 1 (28).

While COPD and RA are comorbidities, their pathological relationship remains under investigation (26, 29). Development of COPD in RA patients (29) is consistent with the known pulmonary involvement in RA (25, 30). Conversely, a recent study identified COPD as a risk factor for RA with the strongest association between COPD and seropositive RA in older smokers (31). Interestingly, individuals who were ACPA positive before RA diagnosis were at increased risk of developing COPD (32). This suggests a linkage between appearance of ACPA and susceptibility to respiratory disease. ACPA may also be an outcome of inhalation of other forms of particulate matter, and may identify inhalant exposures that increase the risk of RA or other autoimmune diseases (33).

### Silica Dust

Exposure to crystalline silica as a result of the breakdown of quartz (e.g. mining, sandblasting, quarrying, ceramics), or during the fabrication of artificial stone (34, 35), can lead to silicosis (36), COPD (36), lung cancer (37), and autoimmune diseases including SLE, RA, SSc, and antineutrophil cytoplasmic antibody (ANCA)-related vasculitis (2, 35, 38). The occurrence of COPD in silica-exposed individuals may also reflect coincident cigarette smoke exposure (39). Significantly, when silica exposure and smoking occur together it results in a synergistic interaction which, for example, greatly increases the risk of ACPA-positive RA in Asian (40) and Caucasian (41) populations. Silica dust exposure is most often *via* occupational inhalation with deposition of respirable particles [<10μm (42)] in the alveoli of the lungs leading to chronic inflammation and development of fibrotic nodules (36). Although the mechanistic aspects of the pathogenic process of silicosis have not been completely defined, a number of important checkpoints have been identified (36, 43, 44). The cumulative dose and physicochemical properties of silica dust are important in inflammatory and fibrogenic responses (43, 45). Inhaled respirable silica particles can be deposited throughout the lung including the distal airways and alveoli leading to direct cytotoxicity (46). Silica particles also interact with endogenous molecules leading to the formation of a surface corona (43). Subsequent phagocytosis by AM leads to lysosomal damage resulting in activation of inflammatory and cell death pathways. A significant contributor to these processes is oxidative stress due to ROS originating from both silica particles and lung cells (47). The chronic persistence of silica particles in the lung results in fibrosis, silicosis (43, 48), and development of silica-induced autoimmunity (49).

An important observation in the initiation of pulmonary inflammation following crystalline silica inhalation is the release of IL-1α from AM following silica induced cellular damage (50). Release of IL-1α from dying cells is known as an important mediator of sterile inflammation (51) following interaction between IL-1α released from crystal damaged cells and IL-1 receptor on surrounding macrophages (48, 50). Silica-induced cell death also results in the release of numerous types of DAMPs that are recognized by TLRs and other PRR on the cell surface as well as within cytoplasmic components such as endosomes. These interactions lead to intracellular signaling, activation of transcription factors such as NF-κB and AP-1, and the expression of inflammatory cytokines such as IL-1β, IL-6, TNF-α, and interferons (IFNs) (48). Phagocytosis of silica particles *via* scavenger receptors such as MARCO (SR-A6) and SR-A1 leads to lysosomal destabilization and release of proteases including cathepsins that contribute to the activation of the NLRP3 inflammasome and cleavage of IL-1β and IL-18 into their bioactive forms (52–54). Generation of ROS, either from the surface of silica crystals or phagocytosis and lysosomal/mitochondrial damage, also contributes to these events (45, 47). The end result is an inflammatory response consisting of neutrophils, monocytes and lymphocytes that exhibits acute and chronic phases which have differing molecular and cellular features (35). The failure to clear silica particles from the lungs exacerbates this chronic inflammatory process leading to fibroblast proliferation and collagen production resulting in fibrosis and silicosis (35, 36, 44).

Epidemiological studies have found associations between occupational silica exposure and SLE, SSc, and ANCA-related vasculitis (2, 55, 56). More recently, exposure to silica-containing dust from artificial stone during fabrication of countertops has been linked to both silicosis and autoimmune disease (34, 57). Pre-clinical features of autoimmunity, such as autoantibodies, as well as autoimmune diseases can occur in the absence of silicosis (35, 55) suggesting that events prior to fibrosis are important for silica-induced autoimmunity. The fibrosis following particulate exposure, including silica, may be the result of exaggerated and persistent immunosuppression due to TGF-β and IL-10, rather than chronic inflammation (58).

A significant outcome of the chronic pulmonary inflammation induced by crystalline silica, in animal models, is the formation of TLS (59). These structures comprise accumulations of B and T cells as well as follicular dendritic cells (FDC) and plasma cells (59–61). The presence of immunoglobulin including autoantibodies in the BALF of silica-exposed mice (60, 61) suggests that the lung is a site of autoantibody production. A variety of autoantibody specificities are found in both humans (62) and experimental models (59, 60, 63) after silica exposure. These include anti-DNA, -SS-A/Ro, -SS-B/La, -Scl70, -Sm, and -RNP. ACPA have also been described in RA patients exposed to silica (40). A possible mechanism for citrullination may involve silica-induced increases in peptidylargininedeiminase (PAD) activity (64). This is consistent with the formation of neutrophil extracellular traps (NETs) during NETosis induced by silica (65) which requires PAD mediated protein citrullination (66). Although autoantibody responses in silica exposed patients with autoimmune diseases are often consistent with those of idiopathic autoimmune diseases, it is unclear if they play a pathogenic role. As with smoking, it is also unclear how expression of silica-induced autoreactivity in the lung results in pathogenesis in organs such as the kidney in SLE or the joints in RA.

## Fibrous Material

### Asbestos

Asbestos describes six naturally occurring fibrous silicate minerals categorized into two groups, serpentine (chrysotile) and amphibole (crocidolite, amosite, tremolite, anthophyllite, actinolite) (67). While there are significant differences in chemical composition and crystalline structure, serpentine fibers are more curvilinear and softer than the needle-like, brittle amphibole fibers (67). Occupational exposures, primarily from mining, construction, and automotive industries, can result in asbestosis and malignancy (68). The properties of asbestos fibers that cause toxicity and pathology, especially inflammation, are uncertain (69), consequently very little is known about the fiber properties necessary for autoimmunity. Short fibers (<5 um), although abundant in the lungs (70), are considered unlikely to cause malignancy (71), but it is unclear if this applies to autoimmunity.

Evidence from both human and experimental animal studies suggest that amphibole asbestos exposure can lead to autoimmunity (4, 72). Several different cohorts including Libby, Montana (73), Wittenoom, Western Australia (74), and Biancavilla, Sicily (75), have linked autoantibodies, including ANA, to asbestos exposure. Studies of the communities of Libby and Troy in Montana have found that almost 14% have been diagnosed with an autoimmune disease other than diabetes (76, 77) and mortality to autoimmune diseases is higher than expected (78). However, a causal role for asbestos exposure in autoimmune disease has not been established due to study limitations (4), and other confounders, including the possibility of concurrent silica and asbestos exposures (2), and smoking history (79).

Asbestos inhalation results in chronic pulmonary inflammation (48), mediated in part by NLRP3 inflammasome activation, and expression of IL-1β and other inflammatory cytokines (80, 81). This is facilitated by asbestos fiber-induced damage to lung epithelial cells, phagocytosis of asbestos particles by macrophages, lysosomal destabilization, subsequent cellular stress and damage leading to production of ROS and oxidative injury, culminating in a fibrogenic response including release of TGF-β, TNF-α, and IL-1β, that promotes collagen deposition and fibrosis (82, 83). Pulmonary inflammation can include increases in AMs and neutrophils, as well as CD4^+^ T cells that spontaneously release IFN-γ, identified as diffuse lymphocytic-macrophage alveolitis (84).

Autoimmune diseases associated with Libby Asbestiform Amphibole (LAA) exposure include SLE, SSc, and RA (76, 78). ANA are common and include autoantibodies against dsDNA, SS-A/Ro52, Scl70, Sm, and RNP (76). Anti-mesothelial cell antibodies, and ANA, have been linked to pleural abnormalities (85). Autoantibodies against cyclic citrullinated peptide (CCP) and RF are not elevated (86) even though there is evidence of increased PAD2/4 and citrullination in the lung following asbestos exposure (87). ANA have also been found in subjects exposed to asbestos without systemic autoimmune diseases (75). This may be an indicator of pre-clinical autoimmunity, similar to the appearance of ACPA prior to COPD or RA (11, 25), as appearance of autoantibodies can precede diagnosis of autoimmune disease (88, 89). Whether autoantibodies can be linked to TLS in the lung in asbestosis, as has been observed for cigarette smoke and crystalline silica exposures, remains to be investigated.

## Nanomaterials

Nanomaterials constitute two major groups, ambient ultrafine particles (UFPs) and ENPs (5, 6, 90). Numerous nanomaterial approaches are being developed to modulate immune responses (91, 92). This includes nanocarriers for drugs, vaccines, antigens and adjuvants (91–93) as therapies for chronic inflammation (91), infection (92), and autoimmunity (91, 92, 94) including specific tolerance approaches for treating autoimmunity (94, 95). The safety of these nanomedicines is dependent upon their ability to avoid toxicity and immune recognition (96, 97) which may result in adverse toxicological (98) and immunological outcomes (99). Although UPFs and ENPs display a range of physicochemical properties (6), inhalation is a common route of exposure (90, 100) and can lead to tissue damage, protein corona, oxidative stress, inflammasome activation, proinflammatory mediators, and inflammation (5, 6, 99, 101), as well as pathological outcomes including fibrosis (58, 102). However, a role for NPs, particularly ENPs, in the causation of pre-clinical human autoimmunity or autoimmune diseases has not been established.

Nanodiamond NPs induce lysosomal damage and NETosis (103) but this leads to resolution of inflammation (103, 104) presumably by sequestration of the offending particles (105). There is evidence for this as NET-related proteins were found in BAL fluid in the acute phase response to TiO_2_ NPs but disappeared over time, and were not associated with histopathological changes (106). Alternatively, solubility or clearance by phagocytes may lead to resolution of NP induced inflammation (103, 107). Nonetheless, nanoparticles appear capable of inducing post-translational protein modification and autoantibodies. Several nanoparticles including, SiO_2_ NPs, cadmium NPs, ultrafine carbon black, and single-wall carbon nanotubes (SWCNT), elicited *in vitro* and/or *in vivo* increases in PAD activity and/or protein citrullination (64, 87, 108), and nickel nanowires stimulated anti-cyclic citrullinated (anti-CCP3) autoantibodies in female C57BL/6 mice (87). However, histological images do not show evidence of pulmonary TLSs following SWCNT exposure (109) or at sites of protein citrullination (64). Thus, although ENPs appear capable of inducing features of experimental pre-clinical autoimmunity (**Table 1**), studies have yet to show that this can mature to autoimmune disease.

**Table 1:** Comparison of hypothesized steps leading to autoimmune disease following exposure to inhaled materials.

Features of Exposure	Silica dust	Cigarette smoke	Asbestos	Nanomaterial
Exposure site	Lung	Lung	Lung	Lung
Inflammatory response components	ROS Inflammasome IL-1α, IL-1β, IL-18, IL-6, TNF-α Interferons NETosis	ROS Inflammasome IL-1β, IL-18, IL-6, TNF-α NETosis	ROS Inflammasome IL-1β, TGF-β, TNF-α, IFN-γ	ROS Inflammasome NETosis
Self-protein citrullination	Yes	Yes	Yes	Yes
Tertiary lymphoid structure in lung	Yes	Yes	Alveolitis	ND
Autoantibodies	ACPA, DNA, SS-A/Ro, SS-B/La, Scl70, Sm, RNP	RF, ACPA	DNA, SS-A/R052, Scl70, Sm, RNP Mesothelial cell	ACPA^X^
Autoimmune diseases associated with exposure	SLE, SSc, RA, ANCA-related vasculitis	Seropositive RA SLE, MS	Rheumatological symptoms of SLE, SSc, RA	ND

Comparison of the hypothesized steps, as described in the text, leading to autoimmune disease following exposure to different inhaled materials. Silica dust and cigarette exposures are linked to the autoimmune diseases indicated. Exposure to asbestos is linked to features of autoimmunity, although evidence to support causation of human autoimmune disease is insufficient. There is no evidence that nanomaterial exposure leads to human autoimmune disease. X, evidence from experimental study only. ND, not determined.

## Discussion

Particulate exposures are among the initiating events most closely linked to human autoimmune diseases (2, 3), with the lungs as a major site of pathological and immunological events leading to clinical disease (26, 27, 33, 110). Such exposures can be protracted and involve particulates resistant to degradation, both of which contributes to the chronic pulmonary inflammation associated with these exposures (48, 79). A complex interplay between pathological processes, including, cellular toxicity, oxidative stress, tissue damage, persistent inflammation, post-translational modification of self-antigens, and the formation of TLSs, promote the generation of autoantibodies that contribute to development of autoimmunity. An unresolved issue is how expression of particulate-induced autoreactivity in the lung results in disease specific pathogenesis in distant organs (e.g. kidney in SLE or joints in RA). However, a recent hypothesis suggests that activated B cells act on preinflamamtory mesenchymal (PRIME) cells which then migrate to the joint in RA (111). The role of pulmonary mesenchymal cells in inflammation (112) supports the possibility of their interaction with B cells in lung TLSs.

Fibrous (asbestos) and non-fibrous (cigarette smoke, crystalline silica) fine (PM_2.5_) particulates have been linked to pre-clinical autoimmunity and autoimmune diseases (2, 76). However, there is little information to support a role for ultrafine (PM_0.1_) particulate matter in human autoimmune disease. This may reflect a difference in size which allows NPs to be cleared from the lung more readily. However, other aspects of pulmonary inflammation including the chronicity and/or resolution of the response, and the apparent absence of TLS, are likely to limit the severity of the adaptive autoimmune response and subsequent development of pathology in target organs other than the lung.

The pathological processes discussed above provide a foundation from which to determine the potential of other particles and fibers to induce autoimmunity. Such studies will provide a better understanding of the physical and chemical properties of particulate matter that lead to the induction and propagation of autoimmune diseases.

## Data Availability Statement

The original contributions presented in the study are included in the article/supplementary material. Further inquiries can be directed to the corresponding author.

## Author Contributions

The author confirms being the sole contributor of this work and has approved it for publication.

## Conflict of Interest

The author declares that the research was conducted in the absence of any commercial or financial relationships that could be construed as a potential conflict of interest
